# Is Preptin a New Bone Metabolism Parameter in Hemodialysis Patients?

**DOI:** 10.3390/life11040341

**Published:** 2021-04-12

**Authors:** Małgorzata Kałużna, Krzysztof Pawlaczyk, Krzysztof Schwermer, Krzysztof Hoppe, Aisha Yusuf Ibrahim, Magdalena Czlapka-Matyasik, Elżbieta Wrotkowska, Katarzyna Ziemnicka, Andrzej Oko, Marek Ruchała

**Affiliations:** 1 Department of Endocrinology, Metabolism and Internal Diseases, Poznan University of Medical Sciences, 60-355 Poznań, Poland; mkaluzna@ump.edu.pl (M.K.); wrotkowska.elzbieta@spsk2.pl (E.W.); kaziem@ump.edu.pl (K.Z.); mruchala@ump.edu.pl (M.R.); 2 Department of Nephrology, Transplantology and Internal Diseases, Poznan University of Medical Sciences, 60-355 Poznań, Poland; kschwermer@ump.edu.pl (K.S.); khoppe@ump.edu.pl (K.H.); aishayusufibrahim@gmail.com (A.Y.I.); aoko@ump.edu.pl (A.O.); 3 Department of Human Nutrition and Dietetics, Poznan University of Life Sciences, 60-624 Poznań, Poland; magdalena.matyasik@up.poznan.pl

**Keywords:** preptin, hemodialysis (HD), end-stage renal disease (ESRD), osteocalcin, dual-energy X-ray absorptiometry (DEXA), chronic kidney disease (CKD), hyperparathyroidism, bone marker

## Abstract

Background: Preptin is a bone-anabolic pancreatic peptide hormone. Its role in bone metabolism has been studied in rats and in patients with diabetes, but its levels and significance in bone metabolism in hemodialyzed (HD) patients is unknown. Methods: The relationships between preptin and anthropometric and biochemical parameters related to bone metabolism were studied in 73 patients on chronic hemodialysis (48 males, 25 females; mean age of 57 years; HD vintage of 69.7 months). Of these subjects, 36 patients had diabetes or impaired glucose tolerance (DM/IGT), and 37 patients had normal glucose tolerance (NGT). Dual-energy X-ray absorptiometry of the femoral neck and lumbar spine were also performed. Results: No differences were observed in preptin levels between DM/IGT and NGT HD patients. Preptin was positively correlated with HD vintage (r = 0.312, *p* = 0.007). Negative correlations between preptin and bone mineral density (BMD), T-score, and Z-score in the lumbar spine (L2-L4) were observed (r = −0.319, *p* = 0.009; r = −0.341, *p* = 0.005; r = −0.375, *p* = 0.002). Preptin was positively correlated with parathormone (PTH) levels (r = 0.379, *p* < 0.001) and osteocalcin levels (r = 0.262, *p* = 0.027). Conclusions: The results indicate that preptin may reflect on bone and mineral metabolism disturbances seen in HD patients. The significant correlation of preptin with PTH and osteocalcin suggests that preptin may be important in indirect measurement of bone turnover in HD patients.

## 1. Introduction

End-stage renal disease (ESRD) patients present with specific bone and mineral metabolism disturbances. Chronic kidney disease-associated mineral and bone disorder accounts for increased morbidity and mortality in those patients. Biochemical markers known thus far do not effectively predict the complex bone changes that are observed in ESRD patients. Diabetes mellitus (DM) itself, the most common cause of ESRD, alters bone metabolism and remodeling [1,2]. Decreased bone mass and lower mineral qualities are usually a combined complication of both DM and ESRD [1,3,4].

Preptin is a pancreatic hormone identified in 2001 by Buchanan et al., which is derived from proIGF-II and co-secreted with insulin. Comprised of 34 amino acids, this bone-anabolic peptide is considered to be an amplifier of glucose-dependent insulin secretion [5]. Plasma preptin levels were found to be higher in patients with type 2 diabetes mellitus (T2DM) [6]. Previous studies showed that preptin could alleviate insulin resistance, as its secretion is associated with increased release of insulin by pancreatic β-cells [3,4,5].

Bone metabolism is systemically regulated by the endocrine system. Recently identified preptin is responsible for the regulation of various human metabolic processes, including bone metabolism. Preptin is believed to be osteogenic in vitro and in vivo [7,8,9]. Studies conducted on animals and human diabetic patients showed that preptin is a bone anabolic hormone [9]. It is involved in bone anabolism in a hyperinsulinemic state [9]. Preptin induces phosphorylation of p42/p44 MAP kinase in osteoblasts and reduces osteoblasts apoptosis, a dose-dependent effect [9]. In mice, preptin enhanced bone area and mineralizing surface [9]. Bosetti et al. observed a modest effect of preptin on human osteoblast activity and differentiation [10]. Serum preptin concentrations were correlated with bone density after adjusting for age and BMI in humans [11]. Furthermore, preptin levels are decreased in osteoporosis and osteopenia in elderly men [11]. To sum up, preptin seems to have stimulating effect on osteoblasts, regulating their proliferation, differentiation, and survival. However, it remains unknown if blood concentrations of preptin are altered in patients with renal failure and on hemodialysis (HD).

The aim of this study is to determine whether preptin could serve as a new bone metabolic parameter in diabetic and non-diabetic hemodialysis patients. Additionally, we aimed to evaluate the relationship between this peptide, body composition, and overhydration in HD patients.

## 2. Materials and Methods

### 2.1. Patients and Methods

The study group consisted of 73 patients treated with maintenance hemodialysis (48 males and 25 females) with an average age of 57 ± 14.5 years and a mean duration of dialysis treatment of 69.7 ± 67.5 months. Of the participants, 36 patients had diabetes or impaired glucose tolerance (DM/IGT), and 37 patients had normal glucose tolerance (NGT). DM/IGT and NGT patients were matched for sex and age. Serum preptin and its relationship to other markers of bone and mineral metabolism were studied in all patients.

All blood samples were collected shortly before the midweek HD session and immediately centrifuged, separated, and frozen at −80 °C. Serum preptin (ng/L) was measured with enzyme immunoassay kits (Sunred Biological Technology Company, Shanghai, China). The sensitivity of the assay was 5.125 ng/L, with the intra-assay coefficient variation of <10%.

Serum glucose (mg/dL) was measured by the hexokinase method. Insulin concentration (ng/mL) was assessed with the DRG Insulin Elisa Kit (analytical sensitivity of 1.76 uIU/mL). Insulin sensitivity was determined by an insulin resistance index (HOMA-IR). HOMA-IR was calculated with the formula: fasting insulin (µU/L) x fasting glucose (mg/dL)/405 [12]. Patients with previously diagnosed DM or HOMA-IR > 3 were included into the DM/IGT group. Parathyroid hormone (PTH, pg/mL) was measured with the ElectroChemiLuminescence Immunoassay (ECLIA, Roche Diagnostics International).

Bone mineral density (BMD) was evaluated by dual-energy X-ray absorptiometry (DEXA) of the femoral neck and lumbar spine (Lunar Prodigy Primo, GE Healthcare). Body composition was assessed by bioimpedance spectroscopy (Body Composition Monitor, Fresenius GmbH, Bad Homburg, Germany). All measurements were conducted prior to the midweek HD session.

### 2.2. Statistical Analysis

Quantitative variables were expressed as medians and interquartile ranges (IQRs). The Shapiro–Wilk test was used to determine whether the data followed a normal distribution. The Mann–Whitney test was performed to identify significant differences between groups. The associations between preptin and other variables were analyzed using Spearman’s correlation coefficient. Statistical analysis was carried out using the StatSoft, Inc. STATISTICA 12; *p*-values < 0.05 were considered statistically significant.

### 2.3. Ethical Standards

All conducted procedures were in accordance with the ethical standards of the responsible committee on human experimentation (institutional and national) and with the Helsinki Declaration of 1975, as revised in 2000. Informed consent has been obtained from all subjects. The study was approved by the Board of Bioethics of Poznan University of Medical Science (587/14; 857/14).

## 3. Results

### 3.1. Patient Characteristics

The clinical characteristics of the subjects are shown in Table 1. In comparison to normal lab values, marked elevations in serum creatinine, osteocalcin, PTH, and phosphates were observed in HD patients (Table 1). No significant difference was observed between the age and HD vintage in DM/IGT and NGT patients (*p* > 0.05). The preptin levels showed high variability among studied patients, 1110.6 ng/L ±1747 (median: 550 ng/L).

### 3.2. NGT and DM-IGT Patients

BMD, T-scores, and Z-scores among NGT and DM/IGT patients were compared. The L2–L4, femoral neck, Ward’s triangle, and total hip BMD, T-score, and Z-score did not statistically differ between these two groups. Furthermore, comparison between NGT and DM/IGT patient groups on HD (Table 2) yielded no notable differences in preptin levels—595 ± 788 ng/L (median: 907.41 ng/L) vs. 512 ± 1030.5 ng/L (median: 1319.44 ng/L), *p* = 0.982. No correlation was found between preptin, glucose, insulin, or HOMA-IR (*p* > 0.05).

### 3.3. Association Between Preptin and Antropometric or HD Parameters

A positive correlation was found between HD vintage and preptin levels in the entire cohort (r = 0.312, *p* = 0.007) and in DM/IGT patients (r = 0.342, *p* = 0.041). There was no correlation between preptin and anthropometric measurements (body mass, height, BMI, waist circumference), age, and body composition measurements assessed by BCM, Fresenius (including lean body mass, fat body mass, overhydration, and extracellular or intracellular water), and parameters of glucose metabolism (*p* > 0.05) (Table 3).

### 3.4. Association Between Preptin and DEXA Results

Negative correlations between preptin and BMD, T-score, and Z-score in the lumbar spine (L2–L4) were observed in the entire cohort (r = −0.319, *p* = 0.010; r = −0.327, *p* = 0.008; r = −0.362, *p* = 0.003, respectively) and in DM/IGT patients (r = −0.423, *p* = 0.014; r = −0.411, *p* = 0.018; r = −0.453, *p* = 0.008, respectively). Furthermore, inverse correlations were found between preptin and Z-score in the femoral neck, Ward’s triangle Z-score, and total hip Z-score in the whole sample (r = −0.241, *p* = 0.049; r = −0.297, *p* = 0.015; r = −0.259, *p* = 0.034, respectively) and in the DM/IGT group (r = −0.505, *p* = 0.002; r = −0.388, *p* = 0.023; r = −0.506, *p* = 0.002, respectively) (Table 4).

### 3.5. Association Between Preptin and Bone and Mineral Metabolism Parameters

Preptin was correlated with PTH (r = 0.379, *p* < 0.001) and osteocalcin levels (r = 0.262, *p* = 0.027) (Table 3). Patients were divided into two groups based on the PTH levels: >200 pg/mL (42 patients) and <200 pg/mL (31 patients). Patients with PTH levels > 200 pg/mL were considered to suffer from clinically significant secondary hyperparathyroidism [13,14,15]. When divided into two groups, the median concentrations of preptin were markedly elevated in patients with PTH > 200 pg/mL—1439.5 ng/L ± 2186.75 (median: 695.5) respectively—as compared to the patients without secondary hyperparathyroidism—665 ng/L ± 658 (median: 452 ng/L), respectively (*p* < 0.05). Patients with PTH levels > 200 pg/mL had higher levels of osteocalcin and alkaline phosphatase (Table 5).

There were no statistically significant correlations between preptin and other parameters, including serum alkaline phosphatase, calcium, and phosphates levels in the two patient subgroups.

## 4. Discussion

Pancreatic β-cell peptide insulin and preptin are proven to be important regulators of bone metabolism [9,10]. This duet of bone-active hormones stimulates anabolic osteoblast activity. Hypersecretion of preptin and insulin stimulates bone formation and inhibits bone resorption [9]. The concentration, function, and importance of preptin in chronic kidney disease–bone mineral disease (CKD-BMD) remains unknown so far. Our study is the first to present preptin levels in HD patients.

High preptin levels were described in newly diagnosed patients with type 2 diabetes (T2DM) [6]. Preptin was independently associated with HOMA-IR in obese and overweight subjects [16]. Elevated preptin levels were also associated with type 1 diabetes (T1DM) and hypertension in T1DM [17]. Furthermore, young women with impaired glucose tolerance had higher preptin concentrations than women with normal glucose tolerance in the study of Bu et al. [18]. Wang et al. observed a correlation between serum preptin concentrations and a higher risk of T2DM and diabetic nephropathy [19]. When comparing preptin concentrations in NGT and DM/IGT patients, no significant differences were observed in the current study. No correlation was observed between preptin and insulin, HOMA-IR, and C-peptide. The lack of a difference between NGT and DM/IGT HD subject groups in our analysis might be the result of renal insufficiency and/or the hemodialysis treatment itself. Insulin and HOMA-IR levels are increased in the initial phase of type 2 diabetes mellitus, and, in patients with long-term diabetes, these indicators could be normal. For this reason, our observations of a lack of correlations between insulin and HOMA-IR and preptin appears to be possible. Subsequent studies on diabetic ESRD patients with subdivision based on the severity and duration of diabetes are needed. Although our data are insufficient in terms of assessing the dialysability of preptin during HD due to no post-HD sampling, a positive correlation between HD vintage and preptin levels seems to suggest that the compound might be accumulated throughout the treatment.

In the studies of Ozkan et al. and Wang et al., preptin levels were increased in patients with a higher BMI [19,20]. Our DM/IGT HD patients had a higher BMI, but there was no correlation between BMI and preptin concentrations. Other variables, such as the presence of IGT, duration and severity of diabetes, and age at diagnosis of diabetes, which were not accounted for in our study, could also play an important role in the evaluation of preptin levels and warrant further investigations. Our DM/IGT group was quite small. Further research evaluating preptin levels in IGT and all types of diabetes are necessary.

The current study also found preptin levels to be negatively correlated with BMD of the lumbar spine region (L2–L4) and the Z-score of Ward’s triangle of the femur neck in ESRD patients. These regions are composed mostly of trabecular bone, so they could be easily affected by general metabolic changes. Our results are inconsistent with literature reports on the anabolic function of preptin in bone. As was mentioned before, preptin has been reported to stimulate the differentiation and proliferation of rat osteoblasts, with no effect on osteoclast activity [9]. Preptin-(1–16), a shorter fragment of preptin, enhances osteoblast proliferation and inhibits osteoblast apoptosis, thus improving the survival rate of primary rat osteoblasts [8,21]. Even shorter fragments of preptin, namely preptin-(1–8), hold the anabolic effect on bone, like the 34 amino acid preptin, and is a potential compound for the development of an oral therapeutic agent in osteoporosis [8]. The effects of preptin on bone formation and pathogenesis of osteoporosis have also been observed in humans [10]. Serum preptin concentrations have been correlated positively with L2-L4, femur neck, and total hip BMD in elderly men in the study of Li et al. [11]. Aahmad et al. observed weak positive correlations between serum preptin levels and femur neck BMD (r = 0.233, *p* = 0.035) and total hip BMD (r = 0.287, *p* = 0.031), but no correlation was captured between preptin and L_1–4_ lumbar spine BMD (r = 0.136, *p* = 0.474) [22]. Our results suggest a potential negative connection between preptin and BMD in HD patients.

The potential negative connection between preptin, PTH, and osteocalcin suggests that preptin might be an important marker in the indirect measurement of bone turnover in HD patients. Blood concentrations of osteocalcin, a bone matrix protein derived from osteoblasts and metabolized in the kidney, are altered in renal failure [23]. Osteocalcin in HD patients is considered as an additional parameter in the diagnosis of severe secondary hyperparathyroidism [23]. A positive correlation between preptin and osteocalcin might support the theory of the potential influence of preptin on bone mineral metabolism in ESRD. In the study of El-Elshmawy et al., serum preptin concentrations were independently associated with osteocalcin in overweight, obese, and normal weight adults, which may underlie the crosstalk between bone and pancreatic β cells [16]. No significant data on the association between preptin and osteocalcin are available in the literature so far.

PTH concentration, a universally used biomarker of bone turnover in clinical practice, correlates with the level of bone turnover in white patients with ESRD [24]. However, PTH lacks specificity and sensitivity in the detailed evaluation of bone turnover [25,26]. There is no evidence in the literature on the relations between preptin and hyperparathyroidism. Preptin concentrations were both related to PTH level and BMD in HD patients in the current study. Possibly, preptin could have a multifactor influence on the bone status in HD patients, and conversely, various mechanisms leading to renal bone disease can affect preptin levels. Renal bone disease is a function of abnormal bone turnover (determined by bone biopsy), bone mineral density (assessed by DXA/quantitative CT), and bone architecture affected by hyperparathyroidism. However, diagnostics of bone status in ESRD remain challenging. Future studies on the relationship between PTH and preptin concentrations should be carried out.

The results indicate that preptin levels could be useful as an additional parameter in hemodialyzed patients with secondary hyperparathyroidism, and could be taken under consideration as additional parameters in the diagnosis of advanced secondary hyperparathyroidism. Preptin might play a role in the pathogenesis of bone mineral disease in ESRD in humans. The effects of preptin on bone metabolism in humans, especially in the case of coexisting diseases like renal insufficiency, have not been thoroughly investigated, and need further research.

The present study has several limitations that need to be considered. The main limitation is the relatively small sample size. A large spread of the preptin values in the study group indicates the need for further investigations in a larger group of CKD patients and ESRD patients treated with different renal replacement therapy. Secondly, NGT and DM/IGT patients were not BMI matched. The BMI of DM/IGT patients was significantly higher than NGT patients. However, no significant associations between preptin and weight and BMI were observed. The cross-sectional design of the present study limits the ability to infer a causal relationship between BMD and preptin concentrations. Studies with repeatable preptin and DXA-derived BMD assessment are needed. Finally, bone histology, the best clinical tool to assess bone turnover, was not used due to invasiveness, and other noninvasive markers had a limited value for assessing bone metabolism. The relation between preptin and bone-specific alkaline phosphatase (BALP) should be evaluated in future studies on patients with ESRD.

## 5. Conclusions

Preptin is being proposed as a new bone metabolic parameter in HD patients. Our results are the first to provide the clinical evidence of the association between preptin and bone metabolism in HD patients. Preptin seems to contribute to the association between β-cells of the pancreas and bones. The results indicate that serum preptin levels could be useful as an additional parameter to assess hemodialyzed patients with secondary hyperparathyroidism. However, this finding needs to be clarified in further studies.

## Figures and Tables

**Table 1:** Baseline characteristic of HD patients.

Parameter	Median (IQR)	Normal Range
Creatinine (mg/dL)	8.35 (3.84)	females: 0.50–0.90; males: 0.70–1.20
Phosphorus (mg/dL)	5.29 (2.34)	2.70–4.50
ALP (U/L)	92.00 (56)	35.00–105.00
PTH (pg/mL)	227.20 (254.70)	15.00–65.00
Osteocalcin (ng/mL)	212.00 (161.00)	24.00–70.00
Preptin (ng/L)	550.00 (917.00)	Not available

ALP—alkaline phosphatase; PTH—parathyroid hormone.

**Table 2:** Baseline and densitometry results in diabetic and non-diabetic HD patients.

Parameter	DM/IGT	NGT	*p*-Value
Age	60.50 (25.00)	55.00 (19.00)	0.608
BMI (kg/m^2^)	27.10 (4.90)	23.30 (5.50)	0.007
HD vintage (months)	45.61 (57.88)	50.63 (65.60)	0.466
Creatinine (mg/dL)	7.835 (4.44)	8.38 (3.50)	0.180
Phosphorus (mg/dL)	5.17 (2.40)	5.97 (2.15)	0.097
ALP (U/L)	91.p00 (49.00)	92.00 (73.00)	0.991
PTH (pg/mL)	225.40 (285.95)	233.00 (186.10)	0.635
Osteocalcin (ng/mL)	191.00 (182.00)	223.50 (127.00)	0.03
Glucose (mg/dL)	94.00 (24.00)	81.00 (17.00)	0.000003
Insulin (µU/mL)	18.95 (12.60)	9.00 (6.00)	<0.000001
HOMA-IR	4.42 (2.965)	1.82 (1.25)	<0.000001
Preptin (ng/L)	512 (1030.50)	595.00 (788.00)	0.982
BMD neck (g/cm^2^)	0.79 (0.126)	0.84 (0.180)	0.704
T-score neck	−1.8 (1.0)	−1.70 (1.4)	0.623
Z-score neck	−0.75 (1.10)	−0.72(0.9)	0.876
Ward’s BMD (g/cm^2^)	0.65 (0.214)	0.68 (0.170)	0.789
Ward’s triangle T-score	−2.1 (1.6)	−2.0 (1.2)	0.818
Ward’s triangle Z-score	−0.65 (1.70)	−0.7 (1.1)	0.562
Total hip BMD (g/cm^2^)	0.83 (0.199)	0.84 (0.171)	0.818
Total hip T-score	−1.55 (1.70)	−1.7 (1.1)	0.818
Total hip Z-score	−0.8 (1.6)	−1.0 (1.0)	0.545
BMD L2–L4 (g/cm^2^)	1.14 (0.293)	1.13 (0.258)	0.616
T-score L2–L4	−0.8 (2.3)	−0.95 (2.05)	0.438
Z-score L2–L4	−0.1 (2.5)	−0.55 (2.20)	0.485

ALP—alkaline phosphatase; BMD—bone mineral density; DM/IGT—patients with diabetes/impaired glucose tolerance; HOMA-IR—insulin resistance index; IQR—interquartile range; L2–L4—second, third, and fourth lumbar vertebrae; NGT—normal glucose tolerance patients. Data are expressed as the median (interquartile range).

**Table 3:** Correlation analysis between preptin levels and anthropometric or laboratory parameters in all studied HD patients, as well as in NGT and DM/IGT subgroups.

Parameter	Preptin (ng/L)		
	All Patients	NGT	DM/IGT
Age	r = −0.199, *p* = 0.089	r = −0.173, *p* = 0.304	r = −0.214, *p* = 0.209
Weight (kg)	r = 0.028, *p* = 0.811	r = −0.023, *p* = 0.888	r = 0.060, *p* = 0.726
BMI (kg/m^2^)	r = 0.037, *p* = 0.754	r = −0.026, *p* = 0.879	r = 0.113, *p* = 0.509
HD vintage (months)	r = 0.312, *p* = 0.007	r = 0.278, *p* = 0.096	r = 0.342, *p* = 0.041
Lean body mass (LBM) (g)	r = −0.020, *p* = 0.856	r = 0.026, *p* = 0.891	r = −0.260, *p* = 0.189
Fat body mass (FBM) (g)	r = 0.066, *p* = 0.550	r = 0.111, *p* = 0.556	r = 0.077, *p* = 0.700
OH (l)	r = −0.160, *p* = 0.141	r = −0.123, *p* = 0.510	r = −0.146, *p* = 0.475
ECW (l)	r = −0.073, *p* = 0.501	r = −0.107, *p* = 0.567	r = −0.265, *p* = 0.181
ICW (l)	r = 0.020, *p* = 0.853	r = 0.169, *p* = 0.365	r = −0.248, *p* = 0.212
ECW/ICW	r = 0.142, *p* = 0.191	r = −0.116, *p* = 0.536	r = −0.088, *p* = 0.662
Creatinine (mg/dL)	r = 0.081, *p* = 0.496	r = −0.090, *p* = 0.595	r = 0.236, *p* = 0.167
Phosphorus (mg/dL)	r = −0.166, *p* = 0.159	r = −0.291, *p* = 0.080	r = −0.113, *p* = 0.511
ALP (U/L)	r = 0.162, *p* = 0.172	r = 0.069, *p* = 0.685	r = 0.257, *p* = 0.130
PTH (pg/mL)	r = 0.379, *p* < 0.001	r = 0.361, *p* = 0.028	r = 0.428, *p* = 0.009
Osteocalcin (ng/mL)	r = 0.262, *p* = 0.027	r = 0.163, *p* = 0.343	r = 0.347, *p* = 0.027
Glucose (mg/dL)	r = −0.042, *p* = 0.726	r = 0.036, *p* = 0.834	r = −0.076, *p* = 0.655
Insulin (µU/mL)	r = −0.024, *p* = 0.840	r = −0.160, *p* = 0.346	r = 0.097, *p* = 0.570
HOMA-IR	r = −0.042, *p* = 0.727	r = −0.127, *p* = 0.454	r = 0.029, *p* = 0.864
C-peptide	r = 0.179, *p* = 0.130	r = 0.085, *p* = 0.615	r = 0.265, *p* = 0.118

ALP—alkaline phosphatase; BMI—body mass index; ECW—extracellular water; HOMA-IR—insulin resistance index; ICW—intracellular water; OH—overhydration; PTH—parathyroid hormone.

**Table 4:** Correlation analysis between preptin levels and densitometry results in all studied HD patients.

Parameter	Preptin (ng/L)		
	Entire Cohort	NGT	DM/IGT
L2–L4 BMD (g/cm^2^)	r = −0.319, *p* = 0.010	r = −0.201, *p* = 0.268	r = −0.423, *p* = 0.014
L2–L4 T-score	r = −0.327, *p* = 0.008	r = −0.234, *p* = 0.197	r = −0.411, *p* = 0.018
L2–L4 Z-score	r = −0.362, *p* = 0.003	r = −0.284, *p* = 0.115	r = −0.453, *p* = 0.008
Femoral neck BMD (g/cm^2^)	r = −0.106, *p* = 0.395	r = 0.073, *p* = 0.685	r = −0.224, *p* = 0.202
Femoral neck T-score	r = −0.109, *p* = 0.378	r = 0.151, *p* = 0.402	r = −0.310, *p* = 0.074
Femoral neck Z-score	r = −0.241, *p* = 0.049	r = 0.093, *p* = 0.606	r = −0.499, *p* = 0.003
Ward’s BMD (g/cm^2^)	r = −0.126, *p* = 0.311	r = 0.026, *p* = 0.885	r = −0.229, *p* = 0.193
Ward’s triangle T-score	r = −0.123, *p* = 0.319	r = 0.112, *p* = 0.536	r = −0.304, *p* = 0.080
Ward’s triangle Z-score	r = −0.297, *p* = 0.015	r = −0.001, *p* = 0.996	r = −0.505, *p* = 0.002
Total hip BMD (g/cm^2^)	r = −0.188, *p* = 0.128	r = 0.027, *p* = 0.883	r = −0.331, *p* = 0.056
Total hip T-score	r = −0.199, *p* = 0.107	r = 0.022, *p* = 0.904	r = −0.388, *p* = 0.023
Total hip Z-score	r = −0.259, *p* = 0.034	r = 0.062, *p* = 0.729	r = −0.506, *p* = 0.002

BMD—bone mineral density.

**Table 5:** Comparison of patients divided on the basis of PTH level.

Parameter	Patients with PTH > 200 pg/mL (*n* = 42)	Patients with PTH < 200 pg/mL (*n* = 31)	*p*-Value
Preptin (ng/L)	695.5 (1184)	452 (579)	*p* = 0.009
Creatinine (mg/dL)	8.42 (4.32)	8.06 (3.32)	*p* = 0.502
Calcium (mg/dL)	9.1 (0.7)	9.1 (0.8)	*p* = 0.727
Phosphorus (mg/dL)	5.26 (2.34)	5.6 (2.58)	*p* = 0.509
Osteocalcin (ng/mL)	230 (96)	137 (178)	*p* = 0.001
ALP (U/L)	103 (64)	75 (32)	*p* = 0.004
L2–L4 BMD (g/cm^2^)	1.11 (0.27)	1.21 (0.39)	*p* = 0.423
L2–L4 T-score	−1.0 (1.9)	−0.3 (3.0)	*p* = 0.317
L2–L4 Z-score	−0.7 (2.1)	0.1 (3.7)	*p* = 0.286
Total hip BMD (g/cm^2^)	0.82 (0.15)	0.89 (0.38)	*p* = 0.271
Total hip T-score	−1.8 (1.1)	−1.1 (2.2)	*p* = 0.083
Total hip Z-score	−1.2 (0.95)	−0.6 (1.4)	*p* = 0.021
Neck BMD (g/cm^2^)	0.78 (0.16)	0.84 (0.32)	*p* = 0.260
Neck T-score	−2.0 (1.2)	−1.6 (2.1)	*p* = 0.106
Neck Z-score	−0.85 (1.25)	−0.5 (0.9)	*p* = 0.013
Ward’s triangle BMD (g/cm^2^)	0.65 (0.16)	0.69 (0.26)	*p* = 0.255
Ward’s triangle T-score	−2.15 (1.35)	−1.9 (1.6)	*p* = 0.101
Ward’s triangle Z-score	−0.85 (1.25)	−0.3 (1.7)	*p* = 0.034

ALP—alkaline phosphatase; BMD—bone mineral density.

## Data Availability

The data that support the findings of this study are available from the corresponding author upon reasonable request.
